# Lower Temperatures Exacerbate NLRP3 Inflammasome Activation by Promoting Monosodium Urate Crystallization, Causing Gout

**DOI:** 10.3390/cells10081919

**Published:** 2021-07-29

**Authors:** Huijeong Ahn, Gilyoung Lee, Geun-Shik Lee

**Affiliations:** College of Veterinary Medicine and Institute of Veterinary Science, Kangwon National University, Chuncheon 24341, Korea; balloon1981@naver.com (H.A.); lky123001@gmail.com (G.L.)

**Keywords:** temperature, NLRP3 inflammasome, gout, monosodium crystals, interleukin-1beta

## Abstract

Gout is a recurrent and chronic form of arthritis caused by the deposition of monosodium urate (MSU) crystals in the joints. Macrophages intake MSU crystals, the trigger for NLRP3 inflammasome activation, which leads to the release of interleukin (IL)-1β and results in the flaring of gout. The effects of temperature, an environmental factor for MSU crystallization, on IL-1β secretion have not been well studied. This study examined the effects of temperature on inflammasome activation. Specific triggers activated canonical inflammasomes (NLRP3, NLRC4, and AIM2) in murine macrophages at various temperatures (25, 33, 37, 39, and 42 °C). The maturation of IL-1β and caspase-1 was measured as an indicator for inflammasome activation. As expected, the optimal temperature of inflammasome activation was 37 °C. The MSU crystal-mediated activation of inflammasome increased at temperatures lower than 37 °C and decreased at higher temperatures. MSU crystals at lower temperatures enhanced IL-1β secretion via the NLRP3 inflammasome pathway. A lower temperature promoted the formation of MSU crystals without changing phagocytosis. Overall, lower temperatures form more MSU crystals and enhance NLRP3 inflammasome activation. In light of these findings, it is possible that hyperthermia therapy may reduce gout flaring.

## 1. Introduction

Gout is a common and chronic form of arthritis caused by the deposition of monosodium urate (MSU) crystals in the articular and connective tissues [1]. Serum urate levels are the crucial risk factor for the development of gout, and hyperuricemia leads to the precipitation of MSU crystals by exceeding the saturation threshold [1,2]. MSU crystals trigger an innate immune response (i.e., NLRP3 inflammasome), resulting in intermittent flaring [2,3]. Thus, an effective treatment of gout is to reduce the concentration of serum urates, which may prevent the deposition of MSU crystals [1]. However, hyperuricemia alone is not considered to be a sufficient condition leading to gout attacks because a large portion of people with hyperuricemia are asymptomatic [1]. The checkpoint for gout flaring is the deposition of MSU crystals, which is influenced by serum urate and other factors, such as temperature, sodium ions, pH, and synovial components [1,2,4].

Inflammasome is a multi-protein complex in myeloid cells and epithelium that leads to an innate immune response by sensing the intracellular danger signals [5]. Uncontrolled and excessive inflammasome activation induces metabolic and degenerative diseases, such as gout, type 2 diabetes, atherosclerosis, heart diseases, inflammatory bowel diseases, and Alzheimer’s disease [5,6]. Canonical inflammasomes are composed of sensor proteins (e.g., NLRP3, NLRC4, and AIM2), ASC protein, and pro-caspase-1 [5,7]. The activation of inflammasome induces caspase (Casp)-1, resulting in cytokine maturation, such as interleukin (IL)-1β and -18, and pyroptosis, a form of inflammatory cell death [5]. Inflammasome is activated through two steps: the priming step mediates the up-regulation of the inflammasome components (e.g., NLRP3 and pro-IL-1β) through the toll-like receptors’ (TLRs) signal pathways, and the activation step induces the formation of inflammasome and activates Casp1 [5,7]. A TLR ligand (e.g., lipopolysaccharide (LPS)) is commonly used as the priming step. Selective triggers for the activation step include the following: nigericin (NG, a bacterial ionophore), monosodium urate (MSU) crystals, and aluminum (alum, an adjuvant) particles for NLRP3; flagellin for NLRC4; and double-strand (ds) DNA for AIM2 [5,8]. Although NLRC4 or AIM2 inflammasomes are activated by cytosolic flagellin or dsDNA, NLRP3 inflammasome activation occurs through complex processes, such as potassium efflux, mitochondrial reactive oxygen species (ROS) production, and cathepsin releases resulting from lysosomal rupture [5,6,7].

Cold therapy is a common recommendation for minor inflammatory diseases (e.g., sprains), as it works by reducing blood flow. In addition, therapeutic hypothermia helps reduce the risk of brain tissue damage caused by a reduced blood supply [9,10]. Thus, temperature may be effective in regulating the initial inflammatory response. To elucidate the effect of temperature on the inflammasome activation, we set temperature variables in consideration of physiological body temperatures [11,12]. Normal body temperature was regarded as 37 °C. The hypothermia and hyperthermia were set at 33 °C and 39 °C, respectively [11]. The temperature of the first metatarsophalangeal joint, where gout attacks are common, is around 32 °C [13]. The foot temperature can drop to room temperature [12]; thus, the lowest temperature was set to 25 °C. The highest temperature was considered to be 42 °C [11]. In this study, temperature was found to regulate inflammasome activation in murine macrophages. Furthermore, the correlation between MSU crystallization and NLRP3 inflammasome activation as a result of temperature changes was elucidated.

## 2. Materials and Methods

Unless indicated otherwise, all materials and information are listed in Supplementary Table S1.

### 2.1. Cell Culture

Bone marrow-derived macrophages (BMDMs) were differentiated from progenitors isolated from the tibias and femurs of C57BL/6 mice using conditioned media containing L929 cell-cultured media (30%, a source of a macrophage colony-stimulating factor), fetal bovine serum (FBS, 10%) and antibiotics at 37 °C in 5% CO_2_ atmosphere. After seven days, the BMDMs were subjected to inflammasome activation. The mice for the BMDMs were approved by the Institutional Animal Care and Use Committee of Kangwon National University (KW-210317-2).

### 2.2. Inflammasome Activation

BMDMs (1 × 10^6^ cells/well in a 12-well-plate) were primed with LPS (1 μg/mL) for 3 h [14]. The LPS-primed BMDMs were replaced with RPMI 1640 media containing the following inflammasome triggers: nigericin (NG, 40 μM) for 1 h; MSU crystals (400 μg/mL) for 3 h; aluminum potassium sulfate (alum, 400 μg/mL) and dsDNA (1 μg/mL) with jetPRIME^TM^ (2 μL/mL) for 1 h; and flagellin (500 ng/mL) with Lipofectamine 2000 (10 μL/mL) for 3 h. The trigger-treated BMDMs were incubated in an incubator set to various temperatures in an atmosphere containing 5% CO_2_. The concentration of CO_2_ was maintained strictly by monitoring with a CO_2_ analyzer to prevent the pH changing, which is another factor contributing to MSU crystallization [2].

### 2.3. Western Blot Analysis

After inflammasome activation, the cellular supernatant (Sup) of the BMDMs were collected, and the cells were lysed using a buffer (1% Triton X-100, 150 mM NaCl, 50 mM Tri-base, pH 8.0) by adding a proteinase inhibitor cocktail. The cellular lysate (Lys) was harvested by centrifugation (15,000 rcf, 5 min), and the remaining debris was treated with suberic acid bis (2 mM) for cross-linking. After 1 h, the pellet (Pellet) was suspended in loading dye for immunoblotting [15].

The samples (i.e., Lys, Sup, and Pellet) were separated by SDS-PAGE (10 or 16%) and transferred to a PVDF membrane using a Mini-PROTEAN Tetra cell system. The membrane was incubated with the following antibodies overnight at 4 °C: anti-Casp1 (p20) antibody, anti-ASC antibody, or anti-Actin antibody. The membrane was further probed with a second anti-sera conjugated with horseradish peroxidase and visualized with a chemiluminescence solution using a chemiluminescent system (EZ-Capture II). The density of the image was analyzed using a CS Analyzer.

### 2.4. ELISA, Phagocytosis, and MSU Solubility Assays

The secretion of IL-1β was analyzed using an ELISA kit. For the phagocytosis rates, BMDMs (1 × 10^4^ cells/well in a 96-well-black plate) were incubated in RPMI 1640 containing 10% FBS, antibiotics, and fluorescent beads at the indicated temperature. After 1 or 6 h, the remaining beads in the media were removed by washing with PBS, and the fluorescence (Ex/Em at 407/505) was measured. For MSU solubility, the MSU crystals were dissolved in distilled water as indicated, and the optical density (OD) from 280 to 330 nm was measured. In addition, MSU crystals were added to RPMI 1640 and incubated at various temperatures (4, 25, 37, 42, and 65 °C). After 3 h, the RPMI 1640 containing MSU was filtered through a syringe filter (with a pore size of 0.45 μm) while maintaining the incubation temperature. The filtered RPMI 1640 was then subjected to OD analysis. All assays were conducted using a microplate spectrophotometer.

### 2.5. Statistical Analyses

Statistical analyses were conducted using GraphPad Prism6. Two groups were analyzed using a Mann–Whitney test and multiple groups were calculated using a one-way non-parametric ANOVA (Kruskal–Wallis test). The significant differences (*p*-values) are marked in the figures.

## 3. Results

### 3.1. Temperature Changes Affect Inflammasome Activation

**Figure 1:**
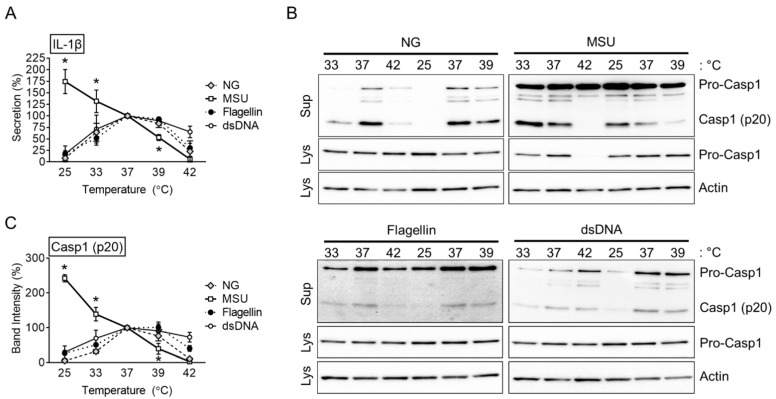
Effects of various temperatures on inflammasome activation. LPS-primed BMDMs were treated with NG, MSU, flagellin, or dsDNA at 25, 33, 37, 39, or 42 °C. (**A**) IL-1β secretion of the BMDMs was measured by ELISA. IL-1β secretion at 37 °C by each trigger was set as 100%, and the graph was drawn. (**B**) Casp1 (p20) secretion was analyzed by immunoblotting. (**C**) The relative intensity of Casp1 (p20) bands are presented as a graph. The graphs present the mean ± standard deviation (SD) of at least two independent experiments, and immunoblotting images are representative of two independent experiments. * *p* < 0.05 vs. NG, flagellin, and dsDNA.

Mouse BMDMs were treated with LPS at 37 °C for 3 h to induce the priming step of inflammasome activation. Subsequently, the cells were incubated separately at different temperatures (25, 33, 37, 39, and 42 °C) for the activation step. The BMDMs were subjected to the NLRP3, NLRC4, or AIM2 triggers. The IL-1β secretion was then measured as an indicator of inflammasome activation. The cells incubated at 37 °C showed the most abundant secretion of IL-1β in response to the NG, flagellin, or dsDNA treatments compared to the other temperatures, as shown in Figure 1A. The level of IL-1β release was reduced if the temperature decreased or increased from 37 °C. On the other hand, the MSU crystal-mediated IL-1β secretion was the highest in the BMDMs at 25 °C and decreased with increasing temperatures. Furthermore, Casp1 (p20) secretion was analyzed by immunoblotting (Figure 1B,C). Similar to the above IL-1β data, Casp1 (p20) secretion from BMDMs treated with the inflammasome triggers (i.e., NG, flagellin or dsDNA) was decreased when the incubation temperature deviated from 37 °C. Nevertheless, the MSU crystals inducing the release of Casp1 (p20) increased with decreasing temperatures from 37 °C and decreased with increasing temperatures. Overall, the incubation at 37 °C was the optimal temperature for the activation of inflammasome activation. In one exception, MSU crystals, an NLRP3 trigger, enhanced inflammasome activation at low temperatures.

### 3.2. MSU Crystals Stimulate More Inflammasome Activation at Lower Temperatures

**Figure 2:**
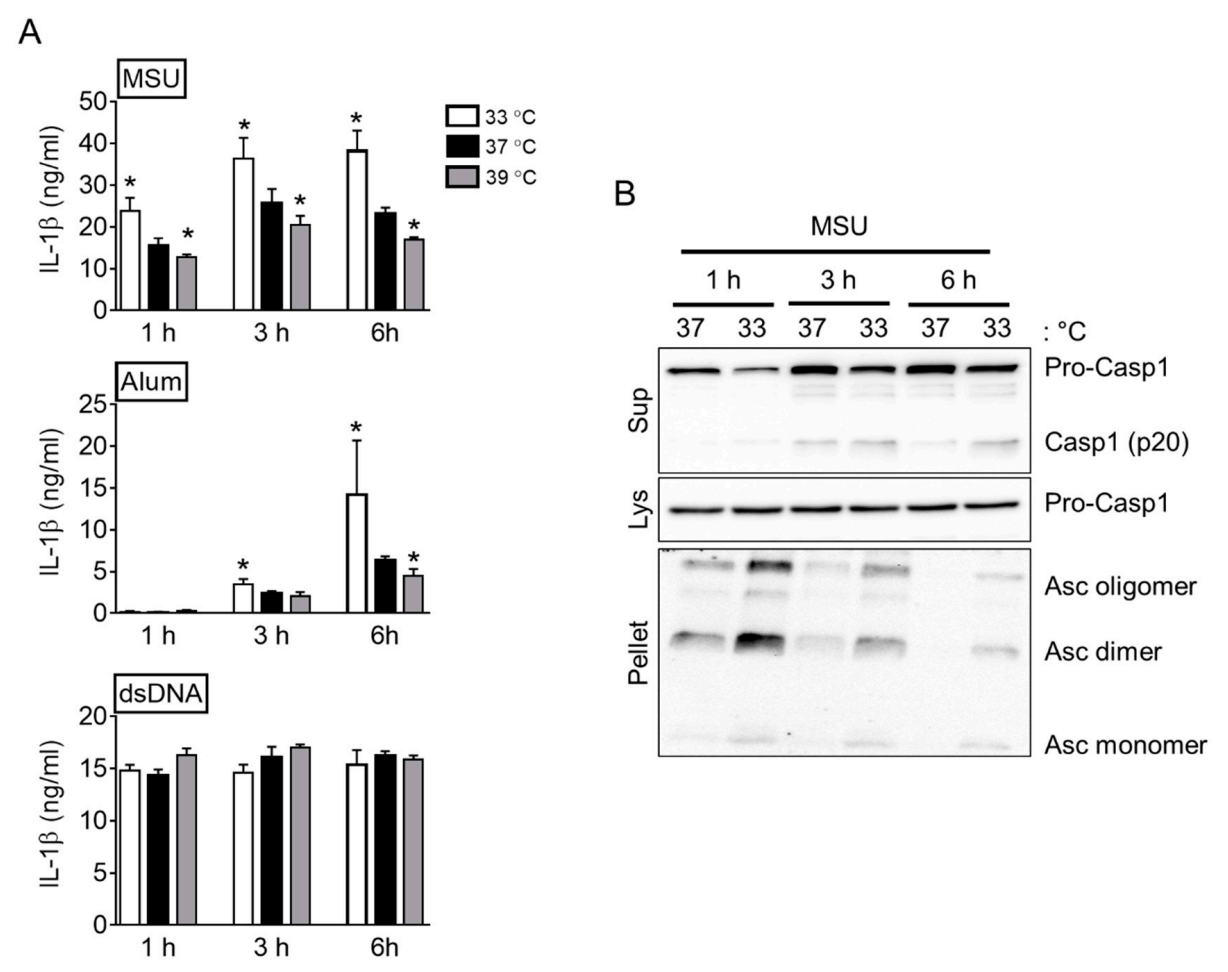
Effect of MSU crystals and alum particles on inflammasome activation at different temperatures. (**A**) LPS-primed BMDMs were treated with MSU crystals, alum particles, or dsDNA at 33, 37, or 39 °C for the indicated time. IL-1β secretion was measured by ELISA. (**B**) LPS-primed BMDMs were treated with MSU crystals and incubated at 33 or 37 °C for 1, 3, or 6 h. The secretion of Casp1 (p20) and the formation of ASC pyroptosome were analyzed by immunoblotting. The bar graphs represent the mean ± standard deviation (SD) of at least two independent experiments, and immunoblotting images are representative of two independent experiments. * *p* < 0.05 vs. 37 °C.

NG and MSU crystals are all NLRP3 triggers, while the active modes differ [5]. NG interacting with pannexin-1 acts as a potassium ionophore to induce NLRP3 inflammasome assembly. On the other hand, MSU crystals are consumed by cells via phagocytosis, which induces NLRP3 inflammasome activation through lysosomal rupture and cathepsin B release. This study elucidated the effects of temperatures on inflammasome activation in the BMDMs treated with MSU crystals or alum particles and found that the effects of NLRP3 occur through phagocytosis. As shown in Figure 2A, IL-1β secretion in response to MSU crystals and alum particles was increased significantly in the BMDM incubated at 33 °C and decreased in the cells at 39 °C compared to that of the cells at 37 °C. In contrast, the IL-1β secretion elicited by the dsDNA treatment was relatively unaffected by changes in temperature. Although the release pattern of IL-1β differed depending on the trigger, the incubating temperature of the LPS-primed BMDMs during the activation step was sufficient to regulate the phagocytosis-mediating inflammasome activation. Furthermore, the formation of ASC pyroptosome, the other readout of inflammasome activation, was observed in response to MSU crystals at 33 or 37 °C (Figure 2B). As expected, the MSU-treated BMDMs at 33 °C showed more Casp1 (p20) secretion and the formation of ASC pyroptosome than that at 37 °C. Overall, a lower temperature might augment inflammasome activation in response to MSU crystals.

### 3.3. Temperature Change Affects the Priming Step of Inflammasome Activation

**Figure 3:**
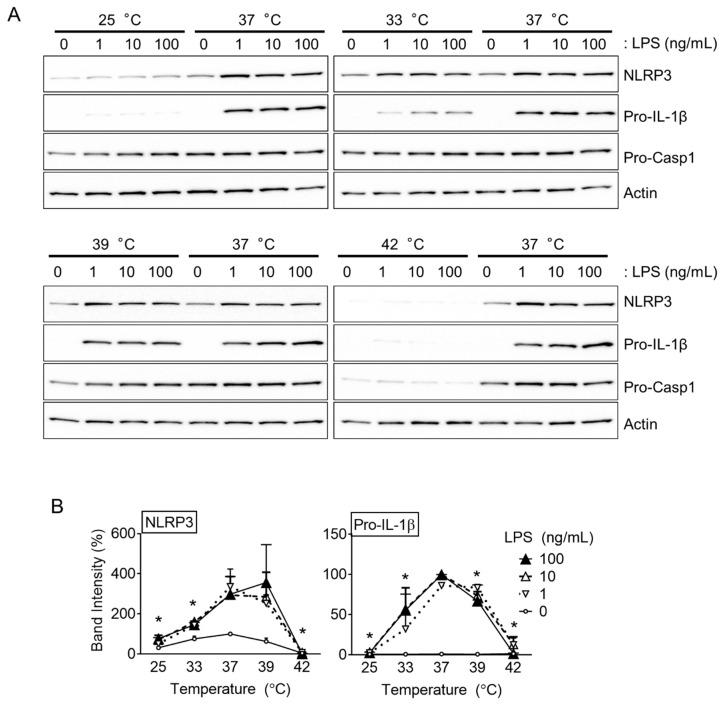
Effects of temperatures on the priming step. BMDMs were treated with increasing dosages of LPS (1, 10, and 100 ng/mL) for 3 h at 25, 33, 37, 39, or 42 °C. (**A**) The levels of NLRP3, pro-IL-1β, and pro-Casp1 expression were determined by immunoblotting. (**B**) The band intensities of NLRP3 and pro-IL-1β were analyzed. The bar graphs present the mean ± standard deviation (SD) of at least two independent experiments, and immunoblotting images are representative of two independent experiments. * *p* < 0.05 vs. 37 °C.

The effects of a temperature change on the priming step of inflammasome activation were examined further. BMDMs were primed with LPS at different temperatures (25, 33, 37, 39, and 42 °C), and the levels of NLRP3, pro-IL-1β, and pro-Casp1 were observed (Figure 3A,B). As a result, all the proteins were expressed abundantly at 37 and 39 °C. On the other hand, the expression of NLRP3 and pro-IL-1β decreased at 33 °C and was barely detectable at 25 and 42 °C. The pro-Casp1 protein was also lower at 42 °C, similar to the others, but it was not tightly regulated by LPS priming. Hence, it was found that incubation at 37 °C was optimal for the priming step, and inflammasome activation may deteriorate at the highest temperature (42 °C).

### 3.4. A Lower Temperature Reinforces NLRP3 Inflammasome Activation in Response to MSU Crystals

**Figure 4:**
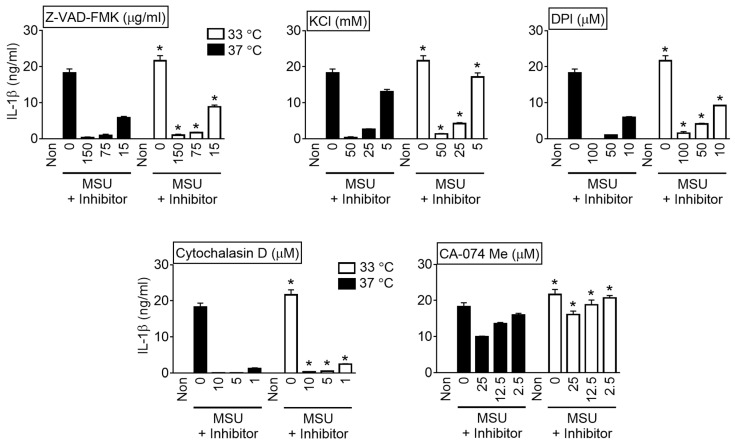
Effect of inhibitors on MSU crystal-mediating inflammasome activation at different temperature. LPS-primed BMDMs activated NLRP3 inflammasome by MSU crystals at 33 or 37 °C in the presence of inhibitors (Z-VAD-FMK, KCl, DPI, cytochalasin D, or CA-074 Me). IL-1β secretion was analyzed by ELISA. The bar graphs represent the mean ± standard deviation (SD) of at least two independent experiments. * *p* < 0.05 vs. 37 °C.

**Figure 5:**
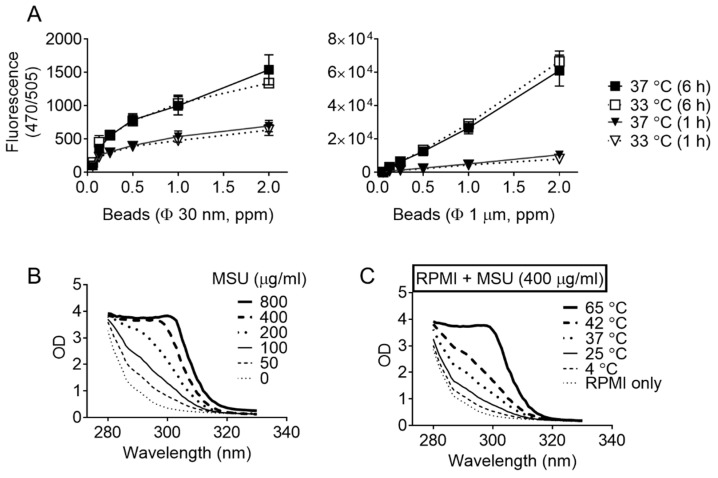
Effects of temperature on phagocytosis and MSU solubility. (**A**) BMDMs were incubated with increasing concentrations of beads at 33 or 37 °C for 1 or 6 h. The phagocytosis rate was analyzed by the fluorescence units inside the cells. (**B**) An increasing concentration of MSU was dissolved in DW and measured OD from 280 to 330 nm. (**C**) MSU (400 μg/mL) was added in PRMI 1640 and incubated at the indicated temperature for 3 h. After eliminating the crystals by filtration, the RPMI 1640 was measured OD from 280 to 330 nm. The graphs present the mean ± standard deviation (SD), and B and C graphs shows the mean of at least two independent experiments.

Several inhibitors of the NLRP3 inflammasome pathway were adopted to determine if the MSU crystals at lower temperatures activated NLRP3 inflammasome (Figure 4). LPS-primed BMDMs were treated with MSU crystals at 33 or 37 °C in the presence of inhibitors. As a result, Z-VAD-FMK (a pan-Casp inhibitor), a KCl solution (a blocker of potassium efflux), DPI (an inhibitor for ROS production), cytochalasin D (a phagocytosis inhibitor), and CA-074 Me (a cathepsin B inhibitors) inhibited IL-1β secretion in a dose-dependent manner [8,16]. On the other hand, the levels of IL-1β differed significantly between temperatures; the IL-1β released at 33 °C was significantly higher than at 37 °C. Next, the effects of temperature on phagocytosis were examined. BMDMs were incubated in the medium containing two sizes of fluorescent beads for 1 or 6 h, and the beads consumed by BMDMs were measured by fluorescence (Figure 5A). As a result, phagocytosis was not altered by temperature. Thus, a lower temperature may enhance IL-1β secretion only via the NLRP3 inflammasome pathway.

### 3.5. Temperature Alters the Formation of MSU Crystals

This study focused on the solubility of MSU depending on temperature. According to the literature [17], the optical density between 280 to 320 nm increased depending on the concentration of the MSU solution in DW (Figure 5B). The MSU crystals were mixed with a medium (i.e., RPMI 1640) in order to trigger NLRP3 inflammasome and were incubated at different temperatures (4 to 65 °C). After 3 h, each MSU mixture was filtered to remove the crystals, and the optical density was measured (Figure 5C). The MSU levels increased with increasing temperatures. As such, a lower temperature formed more crystals. Overall, the lower temperature during the activation step might augment NLRP3 inflammasome activation by forming more MSU crystals.

## 4. Discussion

This study examined the effects of temperature on inflammasome activation in murine macrophages and found that a lower temperature increased the activation of NLRP3 inflammasome in response to MSU crystals, resulting in gout. The optimal temperature of NLRP3, NLRC4, and AIM2 inflammasome activation was 37 °C. The secretion of IL-1β and Casp1 (p20) decreased when the temperature was not optimal. However, MSU crystals did stimulate the secretion of IL-1β and Casp1 (p20) and the formation of ASC pyroptosome at lower temperatures. The incubation at 37 °C was the optimal temperature for the priming step, and the expression of NLRP3 and pro-IL-1β decreased at lower or higher temperatures. MSU crystals at lower temperatures might enhance IL-1β secretion via the NLRP3 inflammasome pathway without altering any other pathway. The phagocytosis of BMDMs remained constant regardless of temperature. Finally, the role of temperature in the solubility of MSU was considered. A higher temperature increased the MSU solubility, while a lower temperature induced the formation of crystals. Overall, the activation of NLRP3 inflammasome triggered by MSU crystals could be regulated by temperature changes. These findings may have applications for curing patients suffering from gout.

Patients with hyperuricemia (beyond 6.8 mg/dL) are prone to gout attacks as a result of increased MSU crystallization [2]. The formation of MSU crystals is influenced by three stages: urate solubility, nucleation, and crystal growth [4]. Although the urea concentration is a key factor in all the stages of MSU crystallization, other factors also regulate each stage: temperature, pH, and the connective tissue factors for the solubility, and the ions and synovial fluid of gout patients, which influence the nucleation [4]. In addition, some gout patients present with normal serum urate levels [2]. Hence, other factors (e.g., temperature) should be considered as potential contributors to gout flaring. Temperature affects the solubility of MSU; a decrease from 37 to 35 °C is sufficient to reduce the solubility of MSU from 6.8 to 6.0 mg/dL [2,17,18]. From a clinical perspective, gout flaring commonly occurs in the first metatarsophalangeal joint, which is the coldest body part [2]. Heat by inflammation may ameliorate gout symptoms by dissolving MSU crystals [2]. Based on these reports and our data, an increase in temperature (e.g., hyperthermia) might alleviate gout attacks by dissolving MSU crystals and reducing inflammation.

In a systemic cohort study of the seasonal incidence and prevalence of gout, it was reported that the number of patients suffering from gout attacks increase during summer [19]. Furthermore, the ambient temperature and humidity are correlated with the risk of gout attacks; hot and dry weather may increase the incidence of gout attacks [20]. Based on these reports, a higher ambient temperature would exacerbate gout attacks, which contrasts with the hypothesis tested in this study. The conflict presented by these results may be accounted for by the following physiological and behavioral changes that occur during hot weather: sweating at higher temperatures, leading to an increase in the serum urate levels resulting from dehydration, and drinking sweetened or alcoholic beverages, a risk factor for gout attacks [20]. Furthermore, the temperature of the distal joints would be lowered by evaporation. Therefore, ambient temperature and the temperature for crystallization should potentially be considered separately in regard to inflammasome activation resulting in gout flaring. Overall, warming attacked joins would reduce the risk of gout attacks by decreasing NLRP3 inflammasome activation.

## Supplementary Materials

The following are available online at https://www.mdpi.com/article/10.3390/cells10081919/s1, Table S1: Details of the materials, original images for blots.
