# Supplementary material for: Lower Temperatures Exacerbate NLRP3 Inflammasome Activation by Promoting Monosodium Urate Crystallization, Causing Gout
Source: Cells. 2021 Jul 29;10(8):1919. doi: 10.3390/cells10081919 (PMC8394355; doi:10.3390/cells10081919)

Supplementary Information

Lower temperature exacerbates NLRP3 inflammasome activation by promoting monosodium urate crystallization, causing gout

Huijeong Ahn†, Gilyoung Lee†, and Geun -Shik Lee*

College of Veterinary Medicine and Institute of Veterinary Science, Kangwon National University, Chuncheon, Gangwon, 24341, Republic of Korea.

Running title: Temperature change regulates gout attack via NLRP3 inflammasome activation

†, These authors contributed equally to this work.

*Correspondence: Geun-Shik Lee, D. V. M., Ph. D.

Laboratory of Inflammatory Diseases, Department of Physiology, College of Veterinary Medicine, Kangwon National University, Chuncheon, Gangwon, 24341, Republic of Korea.

e-mail: leegeun@kangwon.ac.kr, Tel: +82-33-250-8683, Fax: +82-33-244-2367

Supplemental Table S1. The details of materials

Items	Cat. No.	Source
12-well-plate	30012	SPL Life Science, Gyeonggi-do, Republic of Korea
96-well-black plate	30296	SPL Life Science, Gyeonggi-do, Republic of Korea
aluminum potassium sulfate (Alum)	039-4404	Daejung Chemicals & Materials Co. Gyeonggi-do, Republic of Korea
anti-actin antibody	sc-1615	Santa Cruz Biotechnology, Dallas, TX, USA
anti-Asc antibody	sc-22514	Santa Cruz Biotechnology, Dallas, TX, USA
anti-Casp1 (p20) antibody	AG-20B-0042-C100	AdipoGen Co., San Diego, CA, USA
anti-mouse IL-1β antibody	AF-401-NA	R&D Systems, Minneapolis, MN, USA
anti-NLRP3 antibody	AG-20B-0014-C100	AdipoGen Co., San Diego, CA, USA
CA-074 Me	BML-PI126-0001	Enzo Life Science, Inc., New York, NY, USA
chemiluminescence solution (WESTSAVER STAR)	LF-QC0106	AbFrontier, Seoul, Republic of Korea
chemiluminescent system (EZ-Capture II)		ATTO Technology, Tokyo, Japan
CO_2 analyzer (G100)		Geotechnical Instruments, Warwickshire, UK
CS Analyzer (Ver. 3)		ATTO Technology, Tokyo, Japan
cytochalasin D	1233	Tocris Bioscience, Bristol, UK
diphenyleneiodonium (DPI)	0504	Tocris Bioscience, Bristol, UK
fetal bovine serum (FBS)	VWR-97068-085	Avantor, Gyeongsanbuk-do, Republic of Korea
flagellin	tlrl-stfla	Invivogen, San Diego, CA, USA
fluorescent beads	L5155 / L4655	Sigma-Aldrich Co., MO, USA
GraphPad Prism 6		GraphPad Software, San Diego, CA, USA
jetPRIME™	114-01	Polyplus-transfection Inc., Illkirch, France
Lipofectamine 2000	11668027	Invitrogen, Carlsbad, CA, USA
lipopolysaccharide (LPS)	L4130	Sigma-Aldrich Co., MO, USA
mice (C57BL/6)		Nara Biotech, Seoul, Republic of Korea
microplate spectrophotometer (Synergy™ H1)		BioTek, Winooski, VT, USA
mini-PROTEAN Tetra cell system		Bio-Rad, Hercules, CA, USA
Monosodium urate (MSU) crystals	U2875	Sigma-Aldrich Co., MO, USA
mouse IL-1β ELISA kit	DY401	R&D Systems, Minneapolis, MN, USA
nigericin (NG)	4312	Tocris Bioscience, Bristol, UK
potassium solution (KCl)	P2014	Biosesang, Seoul, Republic of Korea
proteinase inhibitor cocktail (Halt™)	78442	ThermoFisher Scientific, Middlesex, MA, USA
PVDF membrane	88518	GE Healthcare Bio-Science, Pittsburgh, PA, USA
RPMI 1640	LM 011-01	Welgene, Gyeongsanbuk-do, Republic of Korea
suberic acid bis	S1885	Sigma-Aldrich Co., MO, USA
Z-VAD-FMK	FMK001	R&D System, Minneapolis, MN, USA

Original Images for Blots of Fig. 1B

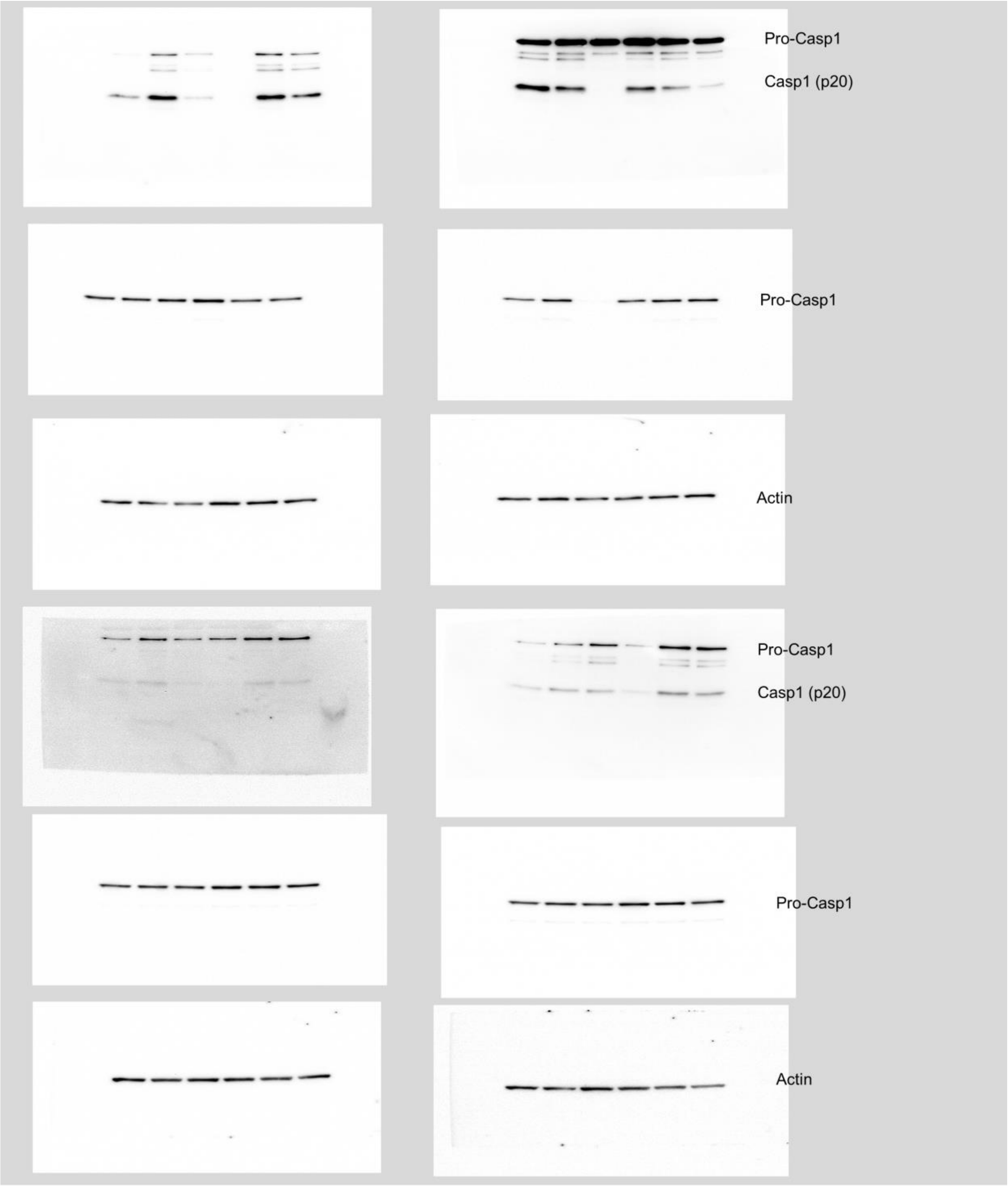

Original Images for Blots of Fig. 2B

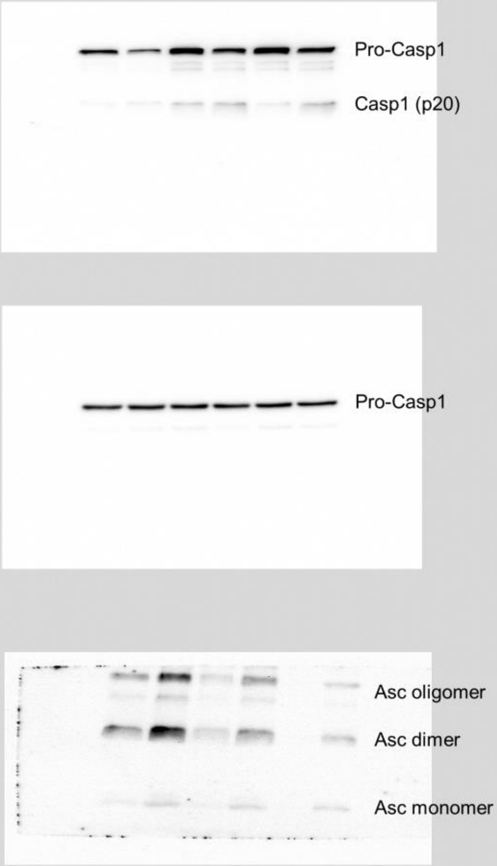

Original Images for Blots of Fig. 3A

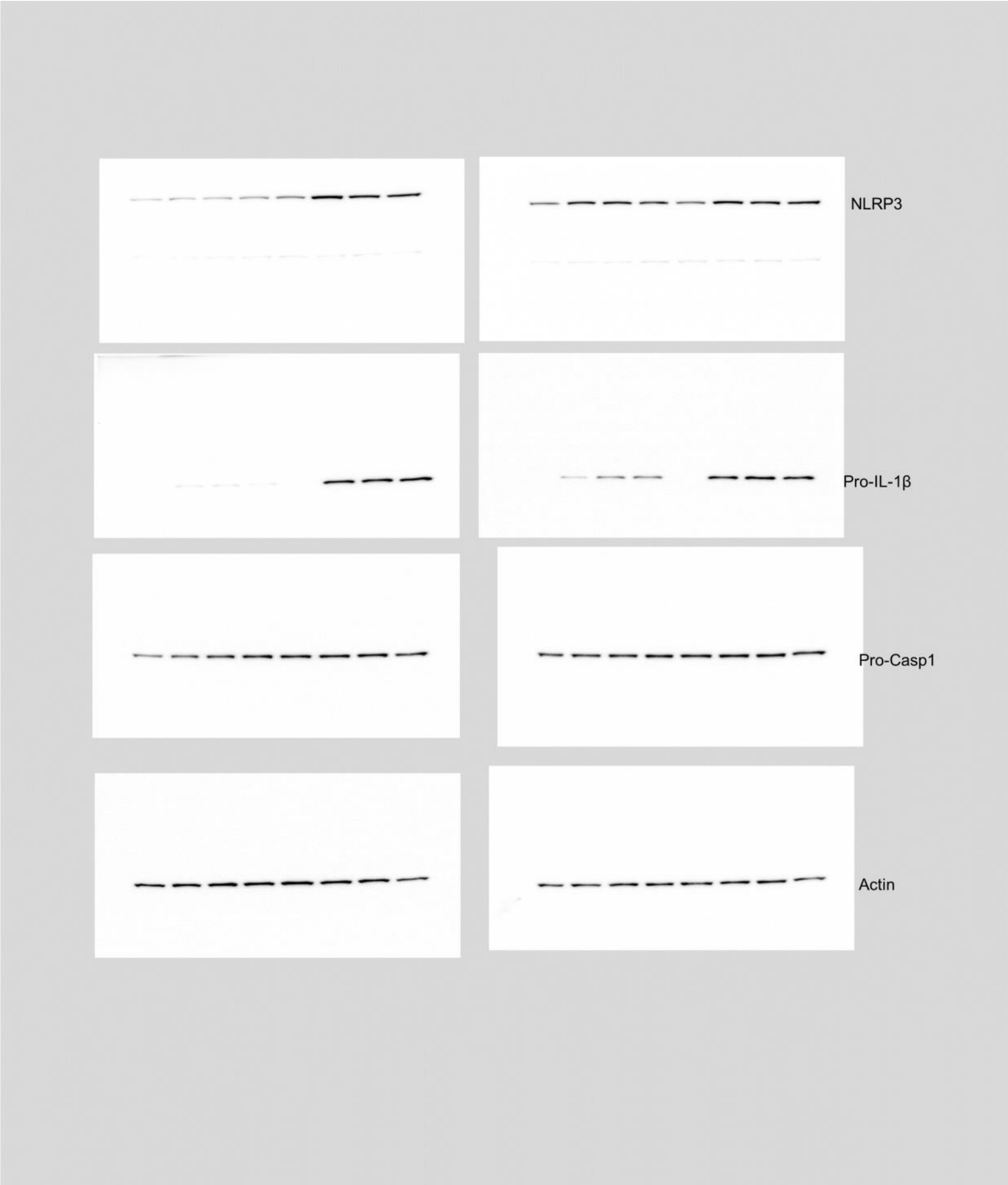

(Continued.) Original Images for Blots of Fig. 3A

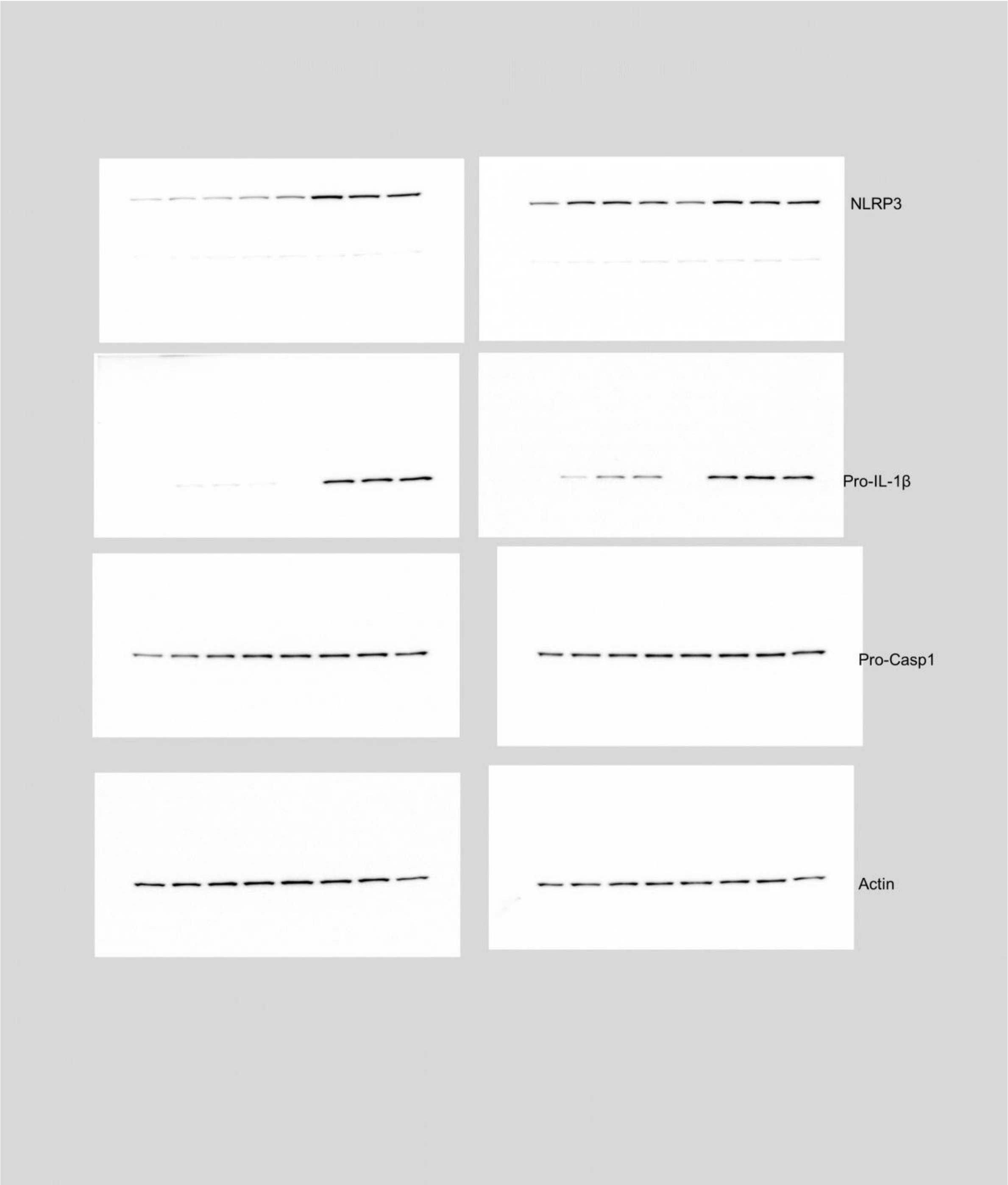

Supplement: Supplementary file 1 [file cells-10-01919-s001.zip › cells-1319228-supplementary.pdf]
